# Transcriptome Dynamics During Turbot Spermatogenesis Predicting the Potential Key Genes Regulating Male Germ Cell Proliferation and Maturation

**DOI:** 10.1038/s41598-018-34149-5

**Published:** 2018-10-25

**Authors:** Xueying Wang, Qinghua Liu, Shihong Xu, Yongshuang Xiao, Yanfeng Wang, Chengcheng Feng, Rui Xue, Haixia Zhao, Zongcheng Song, Jun Li

**Affiliations:** 1 CAS Key Laboratory of Experimental Marine Biology, Institute of Oceanology, Chinese Academy of Sciences, Qingdao, 266071 China; 2 Laboratory for Marine Biology and Biotechnology, Qingdao National Laboratory for Marine Science and Technology, Qingdao, 266071 China; 3 University of Chinese Academy of Sciences, Beijing, 100049 China; 4 Weihai Shenghang Aquatic Product Science and Technology Co. Ltd, Weihai, 264200 China

## Abstract

Spermatogenesis is a dynamic developmental process in which spermatogonial stem cells proliferate, differentiate and mature into functional spermatozoa. These processes require an accurate gene regulation network. Here, we investigated the dynamic changes that occur during spermatogenesis through a combination of histological and transcriptome analyses of different developmental stages of the testis. We constructed 18 testis transcriptome libraries, and the average length, N50, and GC content of the unigenes were 1,795 bp; 3,240 bp and 49.25%, respectively. Differentially expressed genes (DEGs) that were related to germ cell proliferation and maturation, such as *NANOS3*, *RARs*, *KIFs*, steroid hormone synthesis-related genes and receptor genes, were identified between pairs of testis at different developmental stages. Gene ontology annotation and pathway analyses were conducted on DEGs with specific expression patterns involved in the regulation of spermatogenesis. Nine important pathways such as steroid hormone biosynthesis related to spermatogenesis were identified. A total of 21 modules that ranged from 49 to 7,448 genes were designed by a weighted gene co-expression network analysis. Furthermore, a total of 83 candidate miRNA were identified by computational methods. Our study provides the first transcriptomic evidence for differences in gene expression between different developmental stages of spermatogenesis in turbot (*Scophthalmus maximus*).

## Introduction

Fish are the most diverse and abundant group of vertebrates. However, our knowledge about spermatogenesis in teleosts is limited to a few species that are used in basic research and/or aquaculture biotechnology^1^. In general, spermatogenesis is a complex developmental process wherein spermatogonia undergo a mitotic phase (the renewal of spermatogonial stem cells and mitotic proliferation of spermatogonia) and spermatocytes undergo meiosis and differentiate into spermatozoa. In adult testes, the balance between proliferation and differentiation of spermatogonial stem cells is critical for the continuous production of male gametes. Drastic remoulding process occur from the spermatid, the initial haploid cell, to functional spermatozoa. Special structures take shape; the nucleus is condensed, and the cytoplasm and most organelles are discarded during spermiogenesis^2^.

Genes and steroid hormones coordinate the spermatogenesis process. However, detailed information about the genes and pathways that regulate the proliferation and maturation of male germ cells, especially at certain developmental stages, is lacking. During the early spermatogenesis period, mitosis of the spermatogonia and meiosis of the spermatocyte are accompanied by a cytoskeleton rearrangement. At these stages, the genes and pathways involved in cell signalling and cell division, such as *MAPs*, regulate male germ cell proliferation. Steroid hormones, such as progestogens, androgens and oestrogens show important variations during male gonad maturation. They are important regulators of spermatogenesis progression, from spermatogonial stem cell renewal to sperm maturation^1^.

With the rapid development of high-throughput sequencing technologies in recent years, gene expression patterns and their possible physiological functions in specific tissues at specific developmental stages at the transcriptomic level have been able to explored^3^. For example, during human spermatogenesis, transcriptomic dynamics can help predict the potential key genes that regulate male gamete generation^4^; in different boar breeds, transcriptome analyses revealed differences in the development of sexual function^5^ and in yellow catfish, comparative transcriptome analyses highlighted differences in expressed genes and signalling pathways between XY and YY testes^6^. However, information related to spermatogenesis during the different developmental stages in teleosts is limited.

Turbot (*Scophthalmus maximus*, Scophthalmidae, Pleuronectiformes) is an economically important marine economic flatfish species in Europe and China. Recent studies have undertaken the large-scale transcriptome profiling of turbot to identify candidate genes involved in immunity, sex differentiation, sex differences and growth^7–10^. The probable roles of those predicted genes and pathways in turbot reproduction were also discussed. However, the types of male germ cells in the developing testis were different at different stages. Thus, the molecular mechanisms for male germ cell proliferation and maturation needs to be clarified. Further, the gene and signalling pathways for spermatogenesis and spermiogenesis are currently lacking.

The aims of the present study were to assess the transcriptome and the gene expression dynamics of six developmental stages of turbot ranging from MSII to MSVI and to identify pivotal differentially expressed genes (DEGs), gene families, and miRNA that may regulate spermatogenesis. The present study will provide useful information for the exploration of possible spermatogenesis mechanisms at the molecular level for marine fish species.

## Results

### Histological analysis of turbot male germ cells during spermatogenesis

**Figure 1:**
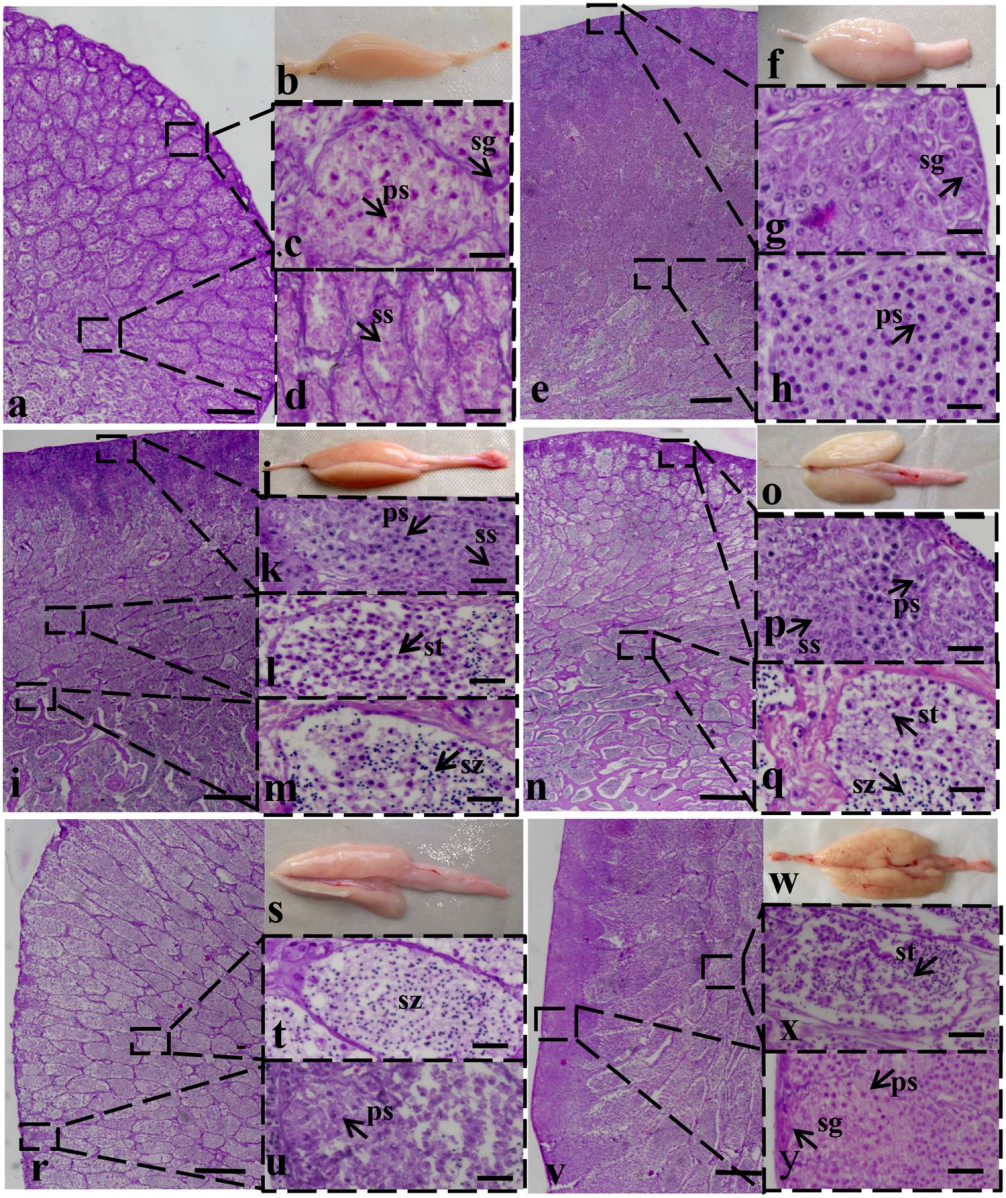
Histological observation of testis at different stages. At stage II (MSII), the major spermatogenic cells were spermatogonium and primary spermatocyte while spermatozoa and spermatid were not found. At stage III, spermatogonia reduced and spermatocyte increased compared to MSII. This is the initiation stage of the annual breeding cycle. Stage III–IV of the testis is the transitional phase. Stage IV is the spermiogenesis stage and there was an increase in the proportion of spermatid. Stage V is the spawning phase and spermatozoa account for the principal parts. Stage VI is testis recession phase, wherein primary spermatocytes were the major cell type. a,b: MSII, e,f: MSIII, i,j: MSIII-MSIV, n,o: MSIV, r,s: MSV, v,w: MSVI. sg, spermatogonium; ps, primary spermatocyte; ss, secondary spermatocyte; st, spermatid; sz, spermatozoon. Scale bars: a,e,i,n,r and v = 500 μm; c,d,g,h,k,l,m,p,q,t,u,x and y = 10 μm.

A haematoxylin and eosin (HE) stain analysis was conducted to understand the proliferation and maturation processes of male germ cells during testis development. Testis tissues were collected at the following different reproductive stages: MSII, MSIII, MSIII-MSIV, MSIV, MSV and MSVI. Morphological observations of the testes and male germ cell structural features are shown in Fig. 1. Spermatogenesis produced cysts and the germ cells of each cyst were in synchronous development. The type and number of germ cells per cyst changed from the MSII to MSVI stages. The histological developmental stage was determined according to the characteristics and ratios of male germ cells. Male germ cell proliferation mainly occurred at MSII and MSIII stages. At the MSII stage, the germ cells included spermatogonium (26.10%) and primary spermatocyte (48.95%) cells. The MSIII stage was the initiation stage of the gonad annual breeding cycle, during which the germ cells demonstrated a higher proliferative activity, including mitosis and meiosis. At this stage, the germ cells were spermatogonium (15.06%), primary spermatocytes (53.87%), secondary spermatocytes (15.03%) and other spermatids (15.87%). At the MSIV stage, most spermatocytes had developed into spermatids, and some of the spermatids had developed into spermatozoa, which then matured and hydrated at the MSV stage. The MSVI stage was the testis recession stage where the germ cell types were mainly primary spermatocyte (63.10%). At the MSVI stage, the ratios of different germ cell types were similar to each other but different compared to the MSIII stage (Table 1).

**Table 1:** Proportion of germ cells in the testis of male turbot collected over an annual cycle.

Germ cell	stage II (%)	stage III (%)	stage III–IV (%)	stage IV (%)	stage V (%)	stage VI (%)
spermatogonium	26.10 ± 2.99 b	15.06 ± 1.76 b	4.50 ± 1.32 d	6.68 ± 1.49 c	1.36 ± 0.25 c	1.52 ± 0.40 d
primary spermatocyte	48.95 ± 6.95 a	53.87 ± 6.13 a	26.59 ± 3.67 b	19.44 ± 1.05 b	14.64 ± 3.85 b	63.10 ± 6.43 a
secondary spermatocyte	24.95 ± 2.37 b	15.03 ± 2.18 b	16.55 ± 1.89 c	6.62 ± 1.16 c	2.42 ± 0.40 c	4.55 ± 1.11 d
spermatid	0.00 ± 0.00 c	15.87 ± 3.23 b	47.63 ± 6.99 a	53.45 ± 6.42 a	16.67 ± 4.82 b	11.32 ± 2.03 c
spermatozoa	0.00 ± 0.00 c	0.00 ± 0.00 c	6.70% ± 0.89 d	3.86 ± 0.37 c	64.33 ± 4.34 a	19.51 ± 1.66 b

Note: The parameters tested were determined using a one-way ANOVA and identified using the Student–Newman–Keuls (SNK) test (P < 0.05). The values are presented as mean ± SD. According to SNK analysis, the letter “a” represents the germ cells proportion whose mean values are relatively larger. From “a” to “d”, the mean values decrease in turn. There is no significant difference with the same letter between different germ cell types in the same stage. There is a significant difference with different letters, (P < 0.05) between different germ cell types in the same stage.

### Sequence analysis and de novo assembly

In this study, 18 cDNA libraries (MSII, MSIII, MSIII-IV, MSIV, MSV and MSVI) were constructed using total RNA from different development stages during spermatogenesis. There were three biological replicates at each stage. To ensure that the RNA-seq data satisfied the criteria for the transcriptome analyses, we conducted standard analyses for quality control. We generated approximately 120.7 Gb bases in total after Illumina HiSeq sequencing. We then produced 132,416 unigenes after assembling all of the samples together. The total length, average length, N50, and GC content of the unigenes were 237,774,222 bp; 1,795 bp; 3,240 bp and 49.25%, respectively. Unigenes were aligned to seven functional databases to obtain possible functional information, and 85,260 (NR database: 64.39%), 103,001(NT: 77.79%), 76,504 (Swissprot: 57.78%), 37,443 (COG: 28.28%), 74,679 (KEGG: 56.40%), 7,804 (GO: 5.89%) and 60,376 (Interpro: 45.60%) unigenes were annotated.

### DEGs at different stages

**Figure 2:**
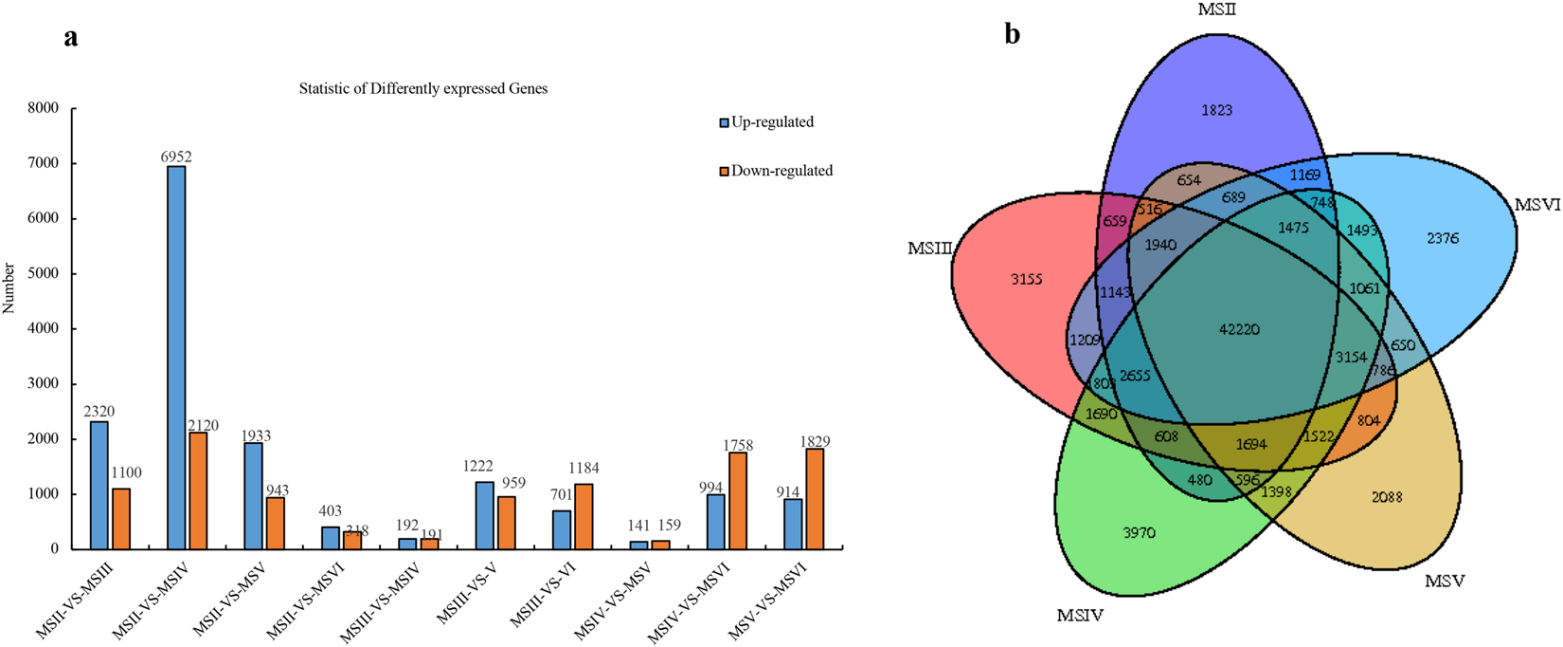
DEGs between the different testis developmental stages. (**a**) Summary of DEGs. The x-axis represents compared samples. The y-axis represents DEG numbers. The blue colour represents up-regulated DEGs and the orange colour represents down regulated DEGs. (**b**) Venn diagram of testicular transcripts from different reproductive phases.

The differential expression of genes at different stages was analysed. The greatest number of DEGs were identified between the MSII and MSIV stages. A total of 6,952 genes were up-regulated and 2,120 genes were down-regulated from MSII to MSIV. Second, in comparisons between MSII vs. MSIII, MSII vs. MSV, MSIV vs. MSVI, MSV vs. MSVI, MSIII vs. MSV and MSIII vs. MSVI, the number of DEGs ranged from 1,885 to 3,420. The number of DEGs was lowest at MSII vs. MSVI, MSIII vs. MSIV and MSIV vs. MSV, with values ranging from 300 to 721 DEGs. A total of 1,222 genes were up-regulated and 959 genes were down-regulated from MSIII to MSV, 141 genes were up-regulated and 159 genes were down-regulated at MSV compared to MSIV, and 914 genes were up-regulated and 1,829 genes were down-regulated from MSV to MSVI (Fig. 2a). A greater number of DEGs were detected between the proliferation and maturation stages than between adjacent development stages. The screened key DEGs between the development stages that were identified in the transcriptome database are listed in Table 2 [log2 (fold) ≥4, P ≤ 0.05]. We identified 1,823 genes that were specifically expressed at the MSII stage, 3,155 at the MSIII stage, 3,970 at the MSIV stage, 2,088 at the MSV stage and 2,376 at the MSVI stage (Fig. 2b).

**Table 2:** Reproduction-related DEGs at different stages.

Gene symbol	Unigene name	E-value	log2 (fold)	Expression quantity	Trend
HSD17B14	Unigene6017_All	4.24E-88	−4.004	II-VS-III	down
IGF1R	CL56.Contig1_All	0	4.894	II-VS-III	up
MNS1	CL28.Contig1_All	0	8.572	II-VS-III	up
CYP20A1	Unigene12025_All	0	5.821	II-VS-III	up
KIF15	CL5909.Contig1_All	0	4.098	II-VS-III	up
NANOS3	Unigene23853_All	1.80E-73	−4.474	II-VS-IV	down
BECN1	CL8838.Contig5_All	0	−5.146	II-VS-IV	down
MNS1	CL28.Contig20_All	0	−5.118	II-VS-IV	down
IGF1R	CL56.Contig4_All	0	8.438	II-VS-IV	up
SPATA17	CL469.Contig5_All	3.50E-71	7.844	II-VS-IV	up
KIF16B	CL12103.Contig19_All	3.01E-70	7.934	II-VS-IV	up
KIF15	CL5909.Contig1_All	0	6.779	II-VS-IV	up
CYP20A1	Unigene16991_All	0	6.241	II-VS-IV	up
SOX13	CL4145.Contig2_All	4.12E-42	4.422	II-VS-IV	up
BMP2	Unigene38766_All	2.00E-58	4.969	II-VS-IV	up
RXRB	Unigene14605_All	0	5.227	II-VS-IV	up
ROPN1B	Unigene38394_All	2.32E-101	5.185	II-VS-IV	up
ATG16	CL8347.Contig5_All	0	4.245	II-VS-IV	up
KIF15	CL5909.Contig3_All	0	−6.901	II-VS-V	down
KIF22	Unigene23278_All	3.12E-124	−5.311	II-VS-V	down
ZP3	Unigene33141_All	1.57E-153	−5.155	II-VS-V	down
KIF16B	CL12103.Contig19_All	3.01E-70	7.104	II-VS-V	up
HSD17B3	CL6671.Contig6_All	1.66E-140	6.342	II-VS-V	up
IGF1R	CL56.Contig4_All	0	6.485	II-VS-V	up
STAR	CL347.Contig2_All	3.07E-156	5.045	II-VS-V	up
ERβ	Unigene6652_All	0	5.568	II-VS-V	up
PGR	CL4345.Contig1_All	2.54E-87	4.590	II-VS-V	up
ROPN1B	Unigene38394_All	2.32E-101	4.544	II-VS-V	up
SPATA17	CL469.Contig5_All	3.50E-71	4.528	II-VS-V	up
SOX13	CL4145.Contig9_All	2.60E-37	4.516	II-VS-V	up
PREB	CL4580.Contig10_All	0	4.332	II-VS-V	up
RXRB	Unigene14605_All	0	4.491	II-VS-V	up
SPAG6	CL10789.Contig6_All	0	4.373	II-VS-V	up
ERβ	Unigene13752_All	0	−5.527	II-VS-VI	down
CYP26A1	Unigene25826_All	0	−4.125	II-VS-VI	down
ZP3	Unigene21371_All	1.50E-160	−4.282	II-VS-VI	down
SPAG6	CL10789.Contig6_All	0	5.404	II-VS-VI	up
OPSIN	CL43.Contig4_All	4.31E-116	4.406	II-VS-VI	up
SOX13	CL4145.Contig9_All	2.60E-37	4.272	II-VS-VI	up
TRAK2	CL11497.Contig17_All	6.33E-34	−5.240	III-VS-IV	down
PANX1	Unigene45806_All	2.39E-178	−4.440	III-VS-IV	down
TUBGCP3	CL382.Contig21_All	2.30E-14	−4.089	III-VS-IV	down
FERMT2	Unigene16518_All	0	4.279	III-VS-IV	up
KIF15	CL5909.Contig3_All	0	−7.484	III-VS-V	down
KIF20B	CL11981.Contig3_All	0	−6.977	III-VS-V	down
MEI1	CL182.Contig17_All	0	−5.924	III-VS-V	down
DNM3	CL12223.Contig9_All	0	−5.454	III-VS-V	down
KIF22	Unigene23278_All	3.12E-124	−5.509	III-VS-V	down
CYP20A1	Unigene10824_All	0	−4.173	III-VS-V	down
IGFBP5	Unigene42773_All	2.85E-138	4.391	III-VS-V	up
WNT2	Unigene26866_All	0	4.341	III-VS-V	up
HSD17B3	CL6671.Contig5_All	3.29E-136	4.308	III-VS-V	up
CYP21A2	CL4229.Contig3_All	9.74E-57	4.214	III-VS-V	up
MNS1	CL28.Contig1_All	0	−7.887	III-VS-VI	down
ATG16	CL8347.Contig5_All	0	−7.172	III-VS-VI	down
SPAG7	CL8142.Contig2_All	5.13E-116	−5.224	III-VS-VI	down
ATG2	Unigene39780_All	3.41E-145	−5.108	III-VS-VI	down
ROPN1	Unigene38391_All	2.32E-101	−4.664	III-VS-VI	down
GZF1	Unigene34367_All	2.62E-16	6.187	III-VS-VI	up
BECN1	CL8838.Contig5_All	0	5.106	IV-VS-V	up
ATG16	CL8347.Contig5_All	0	−7.369	IV-VS-VI	down
ERβ2	Unigene12502_All	0	−6.221	IV-VS-VI	down
ROPN1	Unigene38391_All	2.32E-101	−4.269	IV-VS-VI	down
SOX14	CL4224.Contig1_All	1.05E-116	−4.085	IV-VS-VI	down
MAPK7	CL4998.Contig9_All	0	7.844	IV-VS-VI	up
KIF20B	CL11981.Contig5_All	0	5.589	IV-VS-VI	up
STAR	CL347.Contig2_All	3.07E-156	−4.566	V-VS-VI	down
WNT5	CL9672.Contig4_All	0	−6.190	V-VS-VI	down
PRLR	Unigene46390_All	0	−4.225	V-VS-VI	down
ROPN1B	Unigene38391_All	2.32E-101	−4.160	V-VS-VI	down
KIF15	CL5909.Contig3_All	0	7.692	V-VS-VI	up
KIF22	Unigene23278_All	3.12E-124	5.247	V-VS-VI	up
METTL9	CL9261.Contig7_All	4.12E-31	5.719	V-VS-VI	up

During the process of the testis development cycle, at the MSIII stage, the onset of the spermatogenesis stage, germ cells multiplied greatly. The expression of kinesin and myosin were up-regulated significantly, which regulated the activity of microtubules. Steroid metabolism-related genes such as cytochrome P450 20A1 (*CYP20A1*), thyroid hormone receptor-associated protein 3 (*THRAP3*), oestrogen receptor beta-a (*ERβ-a*) and 17-beta-hydroxysteroid dehydrogenase 14 (*HSD17B14*) were up-regulated significantly in the MSIII stage vs the MSII stage, and played an important role in the onset of spermatogenesis. In addition, the insulin-like growth factor 1 receptor (*IGF1R*), insulin receptor substrate 2-B-like (*IRS2B*), matrix metalloproteinase-15 (*MMP15*), spermatogenesis-associated protein (*SPATA*), autophagy-related protein (*ATG*) and pannexin 1 (*PANX1*) genes were also present at a significantly high expression level in MSIII, suggesting that they may be involved in male germ cell proliferation at spermatogenesis initiation of the annual breeding cycle. At MSV, the sperm mature stage, steroid hormone-related genes and pathways were up-regulated significantly. The *STAR*, testosterone 17-beta-dehydrogenase 3 (*HSD17B3*), steroid 21-hydroxylase (*CYP21A2*), luteinizing hormone receptor (*LHR*), follicle stimulating hormone receptor II (*FSHRII*), stimulated by the retinoic acid gene 6 protein (*STRA6*), prolactin receptor (*PRLR*), steroidogenic factor 1(*NR5A1*) and oestrogen receptor beta genes (*ERβ*), among others, showed obvious high expression levels at the MSV stage. The steroid hormone-related genes and pathways are mainly responsible for regulating the last stage of sperm maturation (Table 2).

### Functional annotation of DEGs

**Figure 3:**
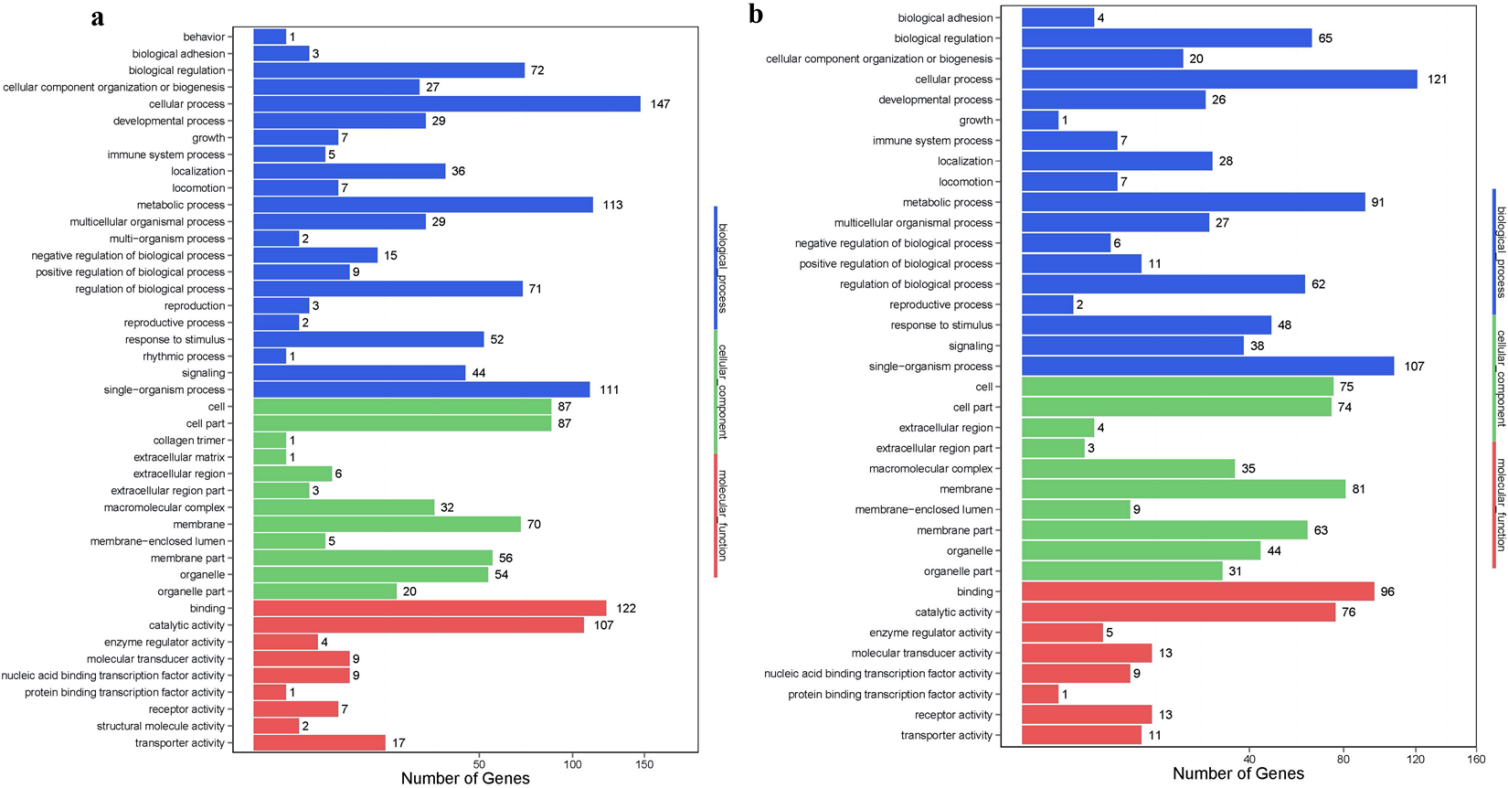
Functional distribution of GO annotation of DEGs between MSII-vs-MSIII and MSIII-vs-MSV. The x-axis represents the number of Unigenes. The y-axis represents the Gene Ontology functional category. (**a**) MSII-vs-MSIII, (**b**) MSIII-vs-MSV.

We performed a gene ontology (GO) classification and functional enrichment on DEGs of different stages. In total, 190 genes that were differentially expressed between the MSII and MSIII stages were assigned molecular functions (180 terms), followed by 174 DEGs that were assigned biological processes (621 terms) and 144 that were assigned cellular components (91 terms) (Fig. 3a). A total of 892 GO terms were assigned to DEGs from the MSII vs MSIII stage comparison. The GO analysis revealed transcripts related to specific processes such as cilium (GO:0005929), cilium organisation (GO:0044782) and Kupffer’s vesicle development (GO:0070121) in MSII vs MSIII. Consistently, DEGs between MSII and MSIII were also enriched in male germ cell proliferation processes such as the regulation of cell division (GO:0051302), regulation of mitotic cell cycle (GO:0007346), regulation of cell proliferation (GO:0042127), germ cell development (GO:0007281), spermatogenesis (GO:0007283) and male gamete generation (GO:0048232). Furthermore, a total of 146 DEGs between MSIII and MSV were assigned molecular functions (189 terms) followed by 146 DEGs assigned biological processes (581 terms) and 133 assigned cellular components (95 terms) (Fig. 3b). In total, 865 GO terms were identified for genes that were differentially expressed between the MSIII and MSV stages. GO analysis between MSIII and MSV uncovered genes related to the cytoskeleton such as microtubule cytoskeleton (GO:0015630), microtubule motor activity (GO:0003777), Kupffer’s vesicle development (GO:0070121), cilium assembly (GO:0042384) and cilium morphogenesis (GO:0060271) genes. GO terms related to steroid metabolism such as steroid hydroxylase activity (GO:0008395), 17-alpha-hydroxyprogesterone aldolase activity (GO:0047442), steroid hormone receptor activity (GO:0003707), progesterone metabolic process (GO:0042448), steroid metabolic process (GO:0008202), steroid hormone mediated signalling pathway (GO:0043401) and cellular response to steroid hormone stimulus (GO:0071383) play important roles in male cell maturation (Supplementary Table S1).

The pathway analysis with the KEGG pathway mapping database provided information on common pathways involved in spermatogenesis. In this study, nine important pathways (steroid hormone biosynthesis, ovarian steroidogenesis, prolactin, TGF-beta, Wnt, GnRH, mTOR, oestrogen and p53 signalling pathways) related to male germ cell proliferation and maturation were identified (Table 3). GO and KEGG annotations were helpful for identifying potential genes related to developmental processes.

**Table 3:** The number of all genes and DEGs annotated in the pathways. The nine pathways are related to turbot spermatogenesis.

pathway	All genes with pathway annotation	DEGs with pathway annotation				
		MSII-vs-MSIII	MSIII-vs-MSIV	MSIII-vs-MSV	MSIV-vs-MSV	MSV-vs-MSVI
Steroid hormone biosynthesis	180	6		19	9	28
Prolactin signalling pathway	517	20	7	29	3	37
TGF-beta signalling pathway	550	22	1	16	3	20
Ovarian steroidogenesis	312	10	7	25	3	40
Wnt signalling pathway	1123	48	2	22	5	37
GnRH signalling pathway	628	21	4	15	4	18
mTOR signalling pathway	369	18		10		14
p53 signalling pathway	518	16		16	1	31
Oestrogen signalling pathway	679	21	2	9	2	16

### Validation of DEGs with quantitative real-time PCR (qRT-PCR)

**Figure 4:**
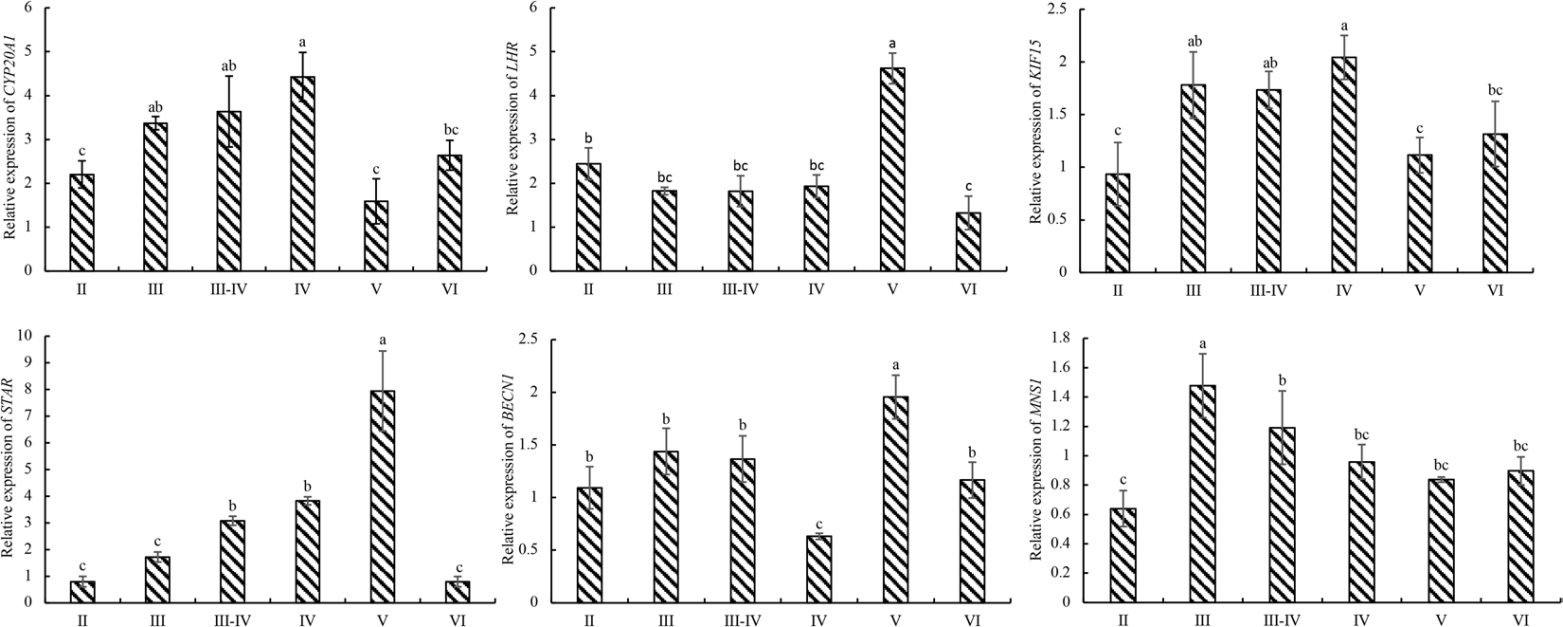
Validation of the DEGs by qRT-PCR. The expression levels of *CYP20A1*, *LHR*, *KIF15*, *STAR*, *BECN1* and *MNS1* during different developmental stages were detected by qRT-PCR. UBQ and RSP were used as reference genes for normalisation of qRT-PCR data. Bars represent the standard deviation (SD). The x-axis indicates the developmental stage. The y-axis shows the relative expression level of genes. Note: The parameters tested were determined using one-way ANOVA and identified using the SNK test (P < 0.05). According to SNK analysis, the letter “a” represents the relative expression levels whose mean values are relatively larger. From “a” to “d”, the mean values decrease in turn. There is no significant difference between stages with the same letter. There is a significant difference between stages with different letters, (P < 0.05).

To evaluate our DEG library, the expression levels of six DEGs, which were primarily involved in spermatogenesis, were analysed by qRT-PCR. Fig. 4 shows the qRT-PCR results, which reflect the same expression trends that were observed through the RNA-seq analysis.

The expression changes in the selected genes (*CYP20A1*, *LHR*, *KIF15*, *STAR*, *BECN1* and *MNS1*) along the reproductive cycle were significantly variable (ANOVA, *P* < 0.05) depending on the reproductive phase. From MSII to MSIII, *CYP20A1*, *KIF15* and *MNS1* were significantly up-regulated, while *LHR*, *STAR* and *BECN1* were significantly up-regulated from MSIII to MSV. These qPCR results showed a similar temporal expression pattern to the one observed following an RNA-seq analysis during the different reproductive phases.

### Co-expression network analysis of DEGs

**Figure 5:**
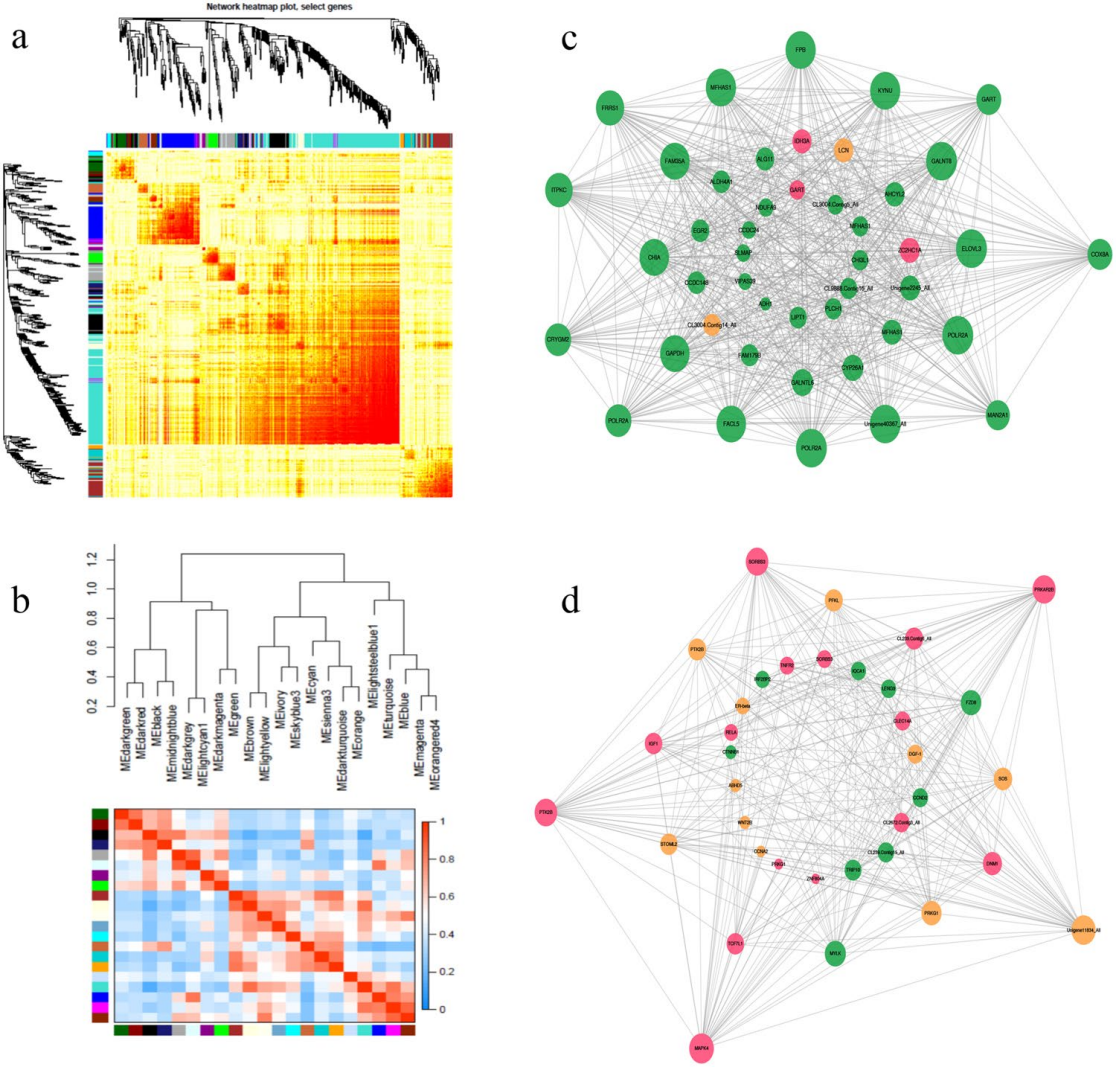
Co-expression network analysis of DEGs. (**a**) The network heatmap of selected genes. Each row and column represents a gene. The light colour indicates a low topological overlap, and a progressively darker colour indicates an increased topological overlap. (**b**) Hierarchical clustering dendrogram of the module eigengenes and a heatmap of the adjacencies using a weighted co-expression network analysis. (**c**) Visualisation of the candidate hub genes from the brown module. (**d**) Visualisation of the candidate hub genes from the black module. Node colour denotes differential expression levels: blue represents down-regulation, red represents up-regulation and orange represents up and down-regulation both presented. Node size represents the importance of a node. The edge denotes the interaction strength.

A weighted gene co-expression network analysis (WGCNA) with scale-free topology was applied to show co-expressed genes across the whole reproductive cycle to systematically and globally identify highly connected gene subnetworks or modules. Groups of genes exhibiting very similar patterns for each module were then detected using hierarchical clustering based on the topological overlap calculations. The expression values of the identified DEGs between different stages were considered, and a total of 21 modules were designed by using different colours in the WGCNA network. Each module had a size ranging from 49 (thistle2 module) to 7,448 genes (red module) (Fig. 5a,b). Furthermore, WGCNA was also used to identify highly correlated genes within these modules. The genes within the modules exhibited more topological overlap than the genes across the modules in the topological overlap heatmap. Modules were individually analysed for the enrichment of GO and KEGG pathways. The terms were screened by extracting those containing the reproduction-related DEGs with a fold change ≥4 and padj ≤0.05 from the modules, which was conducted to provide a biological interpretation of the constructed gene networks. For example, *CYP26A1* from brown module while *ER-β*, *IGF1*, *TRIP10* and *MAPK4* of endocrine system from black module were identified in the gene network visualisation (Fig. 5c,d).

### Identification of the genes involved in spermatogenesis and spermiogenesis

The previously reported genes related to male germ cell proliferation and maturation identified in the present study are listed in Supplementary Tables S2 and S3. Members of the gene families *AQP* (4 isoforms), *SOX* (16 isoforms), and *BMP* (15 isoforms) and *SPATA* (9 isoforms) were widespread and present at high expression levels (Supplementary Table S2). In addition, androgen receptor (*AR*), follicle stimulating hormone receptor (*FSHR*), gonadal soma derived factor (*GSDF*), growth differentiation factor 9 (*GDF9*), meiotic nuclear division protein 1 (*MND1*), anti-Mullerian hormone (*AMH*), *DMRT*, sperm flagellar protein 2 (*SPEF2*), synaptonemal complex protein 3 (*SYCP3*), membrane progesterone receptor alpha (*PAQR7*), progesterone receptor (*PGR*), 20beta-hydroxysteroid dehydrogenase (*HSD20B*) and melatonin (*MLT*) have been previously reported and were also identified in the turbot testis transcriptome (Supplementary Table S3). The genes listed above were all identified in the turbot transcriptome database, though they did not present significantly different expression changes between the different stages, which suggests that they may play multiple roles in regulating the whole spermatogenesis process.

### Turbot miRNA screening from RNA-seq data

A total of 83 candidate miRNAs were identified by computational methods. The sequences and structural properties of known miRNAs were used to screen the candidate miRNAs in the turbot transcriptome databases. The length range of the predicted pm-miRNAs was 18–24 bp. The number of sequences with 22 bp (51.81%) was significantly higher than others (Supplementary Fig. S1). The identified miRNAs are listed in Supplementary Tables S4. Spermatogenesis-related miRNAs, such as miR-34, miR-let-7, miR-29, miR-135, miR-449, miR-203 and so on were identified in the sm-miRNA database (Supplementary Table S4).

## Discussion

In this study, we identified some key genes and pathways regulating spermatogenesis during the reproductive cycle of the testis from different development stages in turbot using histological and RNA-seq analyses. To our knowledge, the present study is the first to investigate spermatogenesis combining male germ cell development with transcriptome in marine fish species. Previous reports related to the process of spermatogenesis in fish mainly focused on morphology descriptions, physiological studies, and the quantity and location of specific genes^11–13^. The large-scale identification of functional genes regulating testis development and spermatogenesis is limited, although omics have been used to investigate biological characteristics such as growth, immunity, sexual dimorphism and sex determination^8–10^. In the present study, we obtained many DEGs between different typical developmental stages. The differences in gene expression between MSII and MSIII, indicate that dramatic changes occurred from spermatogonium proliferation to spermatocytes meiosis. Another significant difference appeared between the MSIII and MSV stages, which indicates that significant changes occurred in the gene network for germ cell development from spermatocytes meiosis to sperm maturation.

At the MSII stage, *NANOS3* (sm-*NANOS3*) was predominantly expressed, with spermatogonium self-renewal and differentiation noted through histological observation. Similar results were obtained in mammals and other fish species^14,15^. For example, in mice, *NANOS3* was expressed in most undifferentiated spermatogonia (As to Aal)^14^, which is responsible for maintaining the undifferentiated state of spermatogonia by the control of their cell cycle^16^. In some teleost, such as common carp (*Cyprinus carpio*)^17^, half-smooth tongue sole (*Cynoglossus semilaevis*)^15^ and zebrafish (*Dania rerio*)^18^, *NANOS3* was also expressed in germline stem cells and maintained the stem characteristics. *NANOS3* might play an important role in regulating the balance of the proliferation and differentiation of spermatogonium cells.

It is well known that RA, which is regulated by the RA synthesis enzymes *ALDH* and degradative enzymes *CYP26*, binds to its receptor to regulate downstream gene expression or acts directly on the effect of gene *STRA8*, regulating the initiation of meiosis during sex differentiation and spermatogenesis in mammals such as mice^19–21^ and some teleost species, such as zebrafish^22^, Nile tilapia (*Oreochromis niloticus*)^23^, and southern catfish (*Silurus meridionalis*)^24^. In turbot, some genes related to RA metabolism and its receptor were significantly up-regulated at the MSIV stage compared with the MSII stage. Meanwhile, by constructing WGCNA gene network for DEGs, *CYP26A1*, one of the degradative cytochrome P450 enzymes, was identified as a candidate brown module hub gene in the gene network visualisation produced by WGCNA analysis. Therefore, we speculated that RA metabolism plays an important role in the regulation of meiosis in turbot by regulating spermatogonial cells during meiosis, including the differentiation of cells to secondary spermatocytes, processes that are observed from stage II to IV. An RA signal pathway is required for germ cell meiotic initiation during spermatogenesis.

In vertebrates, sperm development and maturation are directly regulated by gonadal steroid hormone secretion. The relationship between the expression of genes encoding steroidogenic proteins and receptors for gonadotropins and testicular steroid production have not yet been comprehensively determined in male teleost^25^. In turbot, sperm maturation occurred at the MSV stage, and steroidogenesis enzymes and steroid hormone receptors such as *STAR* and *LHR* both showed obvious high expression levels. Furthermore, members of the ovarian steroidogenesis pathways, such as the *ER* were also significantly up-regulated in turbot at the MSV stage. Oestrogen (E2) has been proven to play an important role in mammals^26^, insects^27^, and fish. Oestrogen significantly influenced the maturation and differentiation of spermatocytes into spermatids^26,28^ and significantly enhanced the sperm fertilising capacity *in vitro*, inducing the acrosome reaction and sperm capacitation^29^ in mammals. For teleost, such as the European eel (*Anguilla Anguilla*)^13^, githead seabream (*Sparus aurata*)^30^, and rainbow darter (*Etheostoma caeruleum*)^31^, *ER* have been reported to be involved in the early stages of spermatogenic cell development. The significant up-regulation of the *ER* and DEG enrichment in the oestrogen pathway at the MSV stage indicated the high importance of E2 in sperm maturation and hydration in turbot. Furthermore, *ER-β*, *IGF1*, *TRIP10* and *MAPK4* of endocrine system were identified in the gene network visualisation from black module. They may experience interaction during sperm maturation.

Kinesins have been reported to be involved in acrosomal biogenesis in the rat^32^, Chinese mitten crab (*Eriocheir sinensis*)^33^, and lobster (*Procambarus clarkia*)^34^. In the present study, members of the kinesin gene family, such as *KIF15*, *KIF16B*, *KIF20B* and *KIF22*, showed significant differences in expression patterns. The kinesins family in turbot might participate in the dynamic cytoskeleton rearrangements of male germ cells, including mitotic and meiotic division, essential organelle transport and the biogenesis of peculiar structures, although no acrosome biogenesis in teleost sperm was observed during male germ cell proliferation, sperm maturation and fertilisation.

In addition, in this study, a total of 83 candidate sm-miRNAs, including miR-34, miR-29, miR-202 and miR-let-7, were identified by bioinformatics combining with four screen criteria. Increasing evidence demonstrates that miRNAs play an important role in various biological processes by negatively regulating target mRNA. In mammals, miR-34 is required by developing gametes and embryos and may be a potential biomarker of sperm quality, miR-202^35^ maintains spermatogonial stem cells by inhibiting cell cycle regulators and RNA binding proteins and miR-29 and miR-let-7^36^ promote SSC differentiation and the meiosis process. Because of their prominent functions, hundreds of miRNAs have been identified in recent years, while only a very small number of miRNAs involved in the spermatogenesis process in teleost have been identified. Therefore, the stage-specific requirements and regulatory mechanism for particular miRNAs in spermatogenesis remain largely uncharacterised for turbot.

In conclusion, transcriptome dynamics during spermatogenesis presented in the present study provide important molecular resources to improve our understanding of the testis development process of turbot. The identification of candidate genes such as *NANOS3*, *RARs*, *KIFs*, steroid hormone synthesis-related genes and receptor genes, related to male germ cells proliferation and maturation of turbot might contribute to find out that their regulatory roles in testis development. In addition, further study of these predicted genes could provide useful insights into turbot male germ cells development.

## Methods

### Experimental fish, and sample collection

The study described in this article was conducted following the European Union (EU) Directive 2010/63/EU for animal experiments (http://ec.europa.eu/environment/chemicals/labanimals/legislation_en.html) and was approved by the ethical committee of the Institute of Oceanology, Chinese Academy of Sciences.

Turbot were reared indoors in circular tanks supplied with flow-through seawater with an ambient temperature of 18 °C ± 0.5 °C and a dissolved oxygen level of ≥5 mg/ml at Shenghang Sci-Tech Co., Ltd. (Weihai, Shangdong Province, China). A photoperiod management regime in accordance with the 8L: 16D, 12L: 12D and 16L: 8D sequential order was used to control gonad development. Three samples were obtained at the same time once in two weeks.

Turbot samples were measured for total weight (±0.001 g), length (±1 mm), and gonad weight (±0.001 g). The gonadosomatic index was 100 (gonad weight/body weight). Gonads were divided in half with one lobe collected and processed for histology and one lobe collected for molecular studies and flash-frozen in liquid nitrogen. The samples were collected from March 2016 to July 2016 to obtain males undergoing distinct developmental stages of the reproductive cycle (Stage–Stage). Stage II testes were collected from 11-month-old males. Approximately 32 males aged between 11 months old (0.38–0.42 kg) and the breeding stage were selected. Testes were excised and examined by anatomical and histological methods to determine their developmental stages.

### Light microscope observation and statistical analysis

A testis lobe from each fish was fixed in Bouin’s solution for 24 h, dehydrated in a series of ethanol, clarified in xylene and embedded in paraffin. The tissues were embedded in paraffin and sectioned with a microtome (Leica RM 2155) at 3 μm thicknesses. All tissue sections were stained with HE. Images were captured by a Nikon Ni-E microscope equipped with a Nikon DS-Ri2 imaging system. All sections from each male fish were examined in a random fashion. Each cell type (spermatogonia, spermatocyte, spermatid, and spermatozoa) at each section of the border and inside of the cyst was scored and five random images per fish were analysed.

### Construction and sequencing of cDNA libraries

Total RNA for each sample was extracted with integrity, and the size distributions of the RNA samples were verified using an Agilent 2200 Bioanalyer (Agilent Technologies, Germany). Samples with an RNA Integrity Number ≥8.0 were used for cDNA library preparation. The concentration of RNA in each extracted sample was measured.

cDNA libraries were constructed for each RNA sample and 18 samples and 18 libraries were sequenced. During the QC step, an Agilent 2100 Bioanalyzer and ABI StepOnePlus Real-Time PCR System were used to quantify and qualify the sample library. Then, the library underwent paired-end sequencing using an Illumina HiSeq X-Ten. After sequencing, we received raw reads and filtered out any reads that had low-quality scores, were adaptor-polluted or had a high content of unknown bases (N) to produce clean reads. The specific parameters for raw reads filtering as follows: remove reads with adaptors; remove reads in which unknown bases(N) comprise >5%; remove low-quality reads (we define a low-quality read as the percentage of base which quality is lesser than 15 is greater than 20% in a read). After filtering, the remaining reads are termed ‘clean reads’. Next, a de novo assembly was run with the clean reads to obtain unigenes.

### Analysis of the transcriptome results

The clean reads were assembled into non-redundant transcripts using the assembler Trinity^37^, which has been developed specifically for the de novo assembly of transcriptomes using short reads. Then, the transcripts were clustered to Unigenes with Tgicl^38^. The Unigenes were used for Basic Local Assignment Search Tool (BLAST) searches and annotation against the NT, NR, COG, KEGG and SwissProt databases^39^. Blast version, v2.2.23; parameters, default; website: http://blast.ncbi.nlm.nih.gov/Blast.cgi. Functional annotation with GO terms (www.geneontology.org) was performed with the Blast2GO software^40^. Blast2GO version, v2.5.0; parameters, default; website: http://www.blast2go.com. The InterPro annotation was performed using InterProScan5 software^41^. InterProScan5 version, v5.11-51.0; parameters, default; website: http://code.google.com/p/interproscan/wiki/Introduction. The clean reads were mapped to the unigenes using *Bowtie2*^42^, and then gene expression levels were calculated with *RSEM*^43^. *Bowtie2* version, v2.2.5; parameters, -q–phred64–sensitive–dpad0–gbar99999999–mp1,1–np1–score-minL,0,-0.1-I1-X1000–no-mixed–no-discordant -p1-k200; website: http://bowtie-bio.sourceforge.net/ *Bowtie2* /index.shtml. *RSEM* version, v1.2.12; parameters, default; website:

http://deweylab.biostat.wisc.edu/ *RSEM*. The gene expression levels were counted as FPKM. We detected DEGs with DEseq2^44^. DEseq2: parameters, fold change ≥2.00 and adjusted P-value ≤ 0.05.

### qRT-PCR validation of the DEGs

Six genes (*CYP20A1*, *LHR*, *KIF15*, *STAR*, *BECN1* and *MNS1*) were chosen for confirmation of DEG data by qRT-PCR. The experiments used SYBR Green (Takara, Dalian, China) detection in a CFX Connect Real-time System (Applied Biosystems, BIORAD, USA). The *UBQ* and *RSP* genes were used as reference genes in these experiments^45^. Relative gene expression data were analyzed using the 2^−ΔΔCt^ method. All reactions were carried out in triplicate.

### WGCNA

A gene co-expression network was constructed using the WGCNA method, which was implemented with the WGCNA package in R^46^.

### Reproduction-related genes during spermatogenesis identified from transcriptome sequences

The identification of homologous unigenes with spermatogenesis-related proteins was carried out by searching the key words of reported proteins in BLASTX alignment results with the databases mentioned above. The identification of key unigenes that regulate male germ cell proliferation and maturation during different development stages was based on the differential expression results.

### Bioinformatics prediction of miRNAs from transcriptome sequences

Sequences used for sm-miRNA prediction were derived from a local BLAST search that compared the turbot transcriptome database with the miRNA hairpin database. The procedures for searching for candidate sm-miRNA were performed, as previously reported, with some modification (evalue = 1e-5)^47^.

## Electronic supplementary material

Supplementary information

## Data Availability

Sequence data generated for the present study has been deposited to NCBI Short Read Archive. The SRA ID assigned is: SRP136753.
