# Supplementary material for: Transcriptome Dynamics During Turbot Spermatogenesis Predicting the Potential Key Genes Regulating Male Germ Cell Proliferation and Maturation
Source: Sci Rep. 2018 Oct 25;8:15825. doi: 10.1038/s41598-018-34149-5 (PMC6202422; doi:10.1038/s41598-018-34149-5)
Supplement: Supplementary file 1 — Supplementary information [file 41598_2018_34149_MOESM1_ESM.pdf]

Transcriptome Dynamics During Turbot Spermatogenesis Predicting the Potential Key Genes Regulating Male Germ Cell Proliferation and Maturation

Xueying Wang[1, 2#], Qinghua Liu[1, 2#,*], Shihong Xu[1, 2], Yongshuang Xiao[1, 2], Yanfeng Wang[1, 2], Chengcheng Feng[1, 2, 3], Rui Xue[1, 2, 3], Haixia Zhao[1, 2, 3], Zongcheng Song[4], Jun Li [1, 2,*]

Supplementary Table S1. The list of reproduction-related DEGs in GO annotation between different developmental stages.

Terms	Gene Ontology term	GO
MSII-VS-MSIII.DEseq2_Method_C	Myosin complex	GO:0016459
	cilium	GO:0005929
MSII-VS-MSIII.DEseq2_Method_P	nonmotile primary cilium assembly	GO:0035058
	regulation of mitotic sister chromatid separation	GO:0010965
	regulation of metaphase/anaphase transition of cell cycle	GO:1902099
	regulation of mitotic nuclear division	GO:0007088
	cilium assembly	GO:0042384
	spermatid development	GO:0007286
	spermatid differentiation	GO:0048515
	cilium organization	GO:0044782
	cilium movement	GO:0003341
	Kupffer's vesicle development	GO:0070121
	regulation of chromosome organization	GO:0033044
	regulation of cell division	GO:0051302
	cilium morphogenesis	GO:0060271
	regulation of mitotic cell cycle phase transition	GO:1901990
	cell morphogenesis involved in differentiation	GO:0000904
	regulation of cell proliferation	GO:0042127
	germ cell development	GO:0007281
	spermatogenesis	GO:0007283
	male gamete generation	GO:0048232
	regulation of mitotic cell cycle	GO:0007346
	regulation of cell cycle process	GO:0010564
	positive regulation of cell migration	GO:0030335
	cellular lipid metabolic process	GO:0044255

	gamete generation	GO:0007276
MSIII-VS-MSV.DEseq2_Method_C	microtubule cytoskeleton	GO:0015630
	myosin complex	GO:0016459
	actin cytoskeleton	GO:0015629
MSIII-VS-MSV.DEseq2_Method_F	steroid hydroxylase activity	GO:0008395
	17-alpha-hydroxyprogesterone aldolase activity	GO:0047442
	motor activity	GO:0003774
	microtubule motor activity	GO:0003777
	steroid hormone receptor activity	GO:0003707
	lipid binding	GO:0008289
MSIII-VS-MSV.DEseq2_Method_P		
	C21-steroid hormone metabolic process	GO:0008207
	progesterone metabolic process	GO:0042448
	steroid biosynthetic process	GO:0006694
	formation of primary germ layer	GO:0001704
	steroid metabolic process	GO:0008202
	cellular hormone metabolic process	GO:0034754
	cell surface receptor signaling pathway	GO:0007166
	regulation of cell division	GO:0051302
	positive regulation of cell proliferation	GO:0008284
	cell cycle checkpoint	GO:0000075
	lipid metabolic process	GO:0006629
	lipid biosynthetic process	GO:0008610
	Kupffer's vesicle development	GO:0070121
	hormone metabolic process	GO:0042445
	photoreceptor cell development	GO:0042461
	Wnt signaling pathway	GO:0016055
	regulation of cell cycle process	GO:0010564
	steroid hormone mediated signaling pathway	GO:0043401
	cellular response to steroid hormone stimulus	GO:0071383
	regulation of hormone levels	GO:0010817
	cell motility	GO:0048870
	response to lipid	GO:0033993
	microtubule cytoskeleton organization	GO:0000226
	regulation of cell proliferation	GO:0042127
	lipid transport	GO:0006869
	lipid localization	GO:0010876
	cilium organization	GO:0044782
	mitotic cell cycle	GO:0000278
	cilium assembly	GO:0042384
	mitotic cell cycle process	GO:0044255

	reproductive process	GO:0022414
	reproduction	GO:0000003
	cilium morphogenesis	GO:0060271
MSIV-VS-MSV.DEseq2_Method_C	cytoskeletal part	GO:0044430
	cytoskeleton	GO:0005856
MSIV-VS-MSV.DEseq2_Method_P	response to light stimulus	GO:0009416
MSIV-VS-MSVI.DEseq2_Method_C	cilium	GO:0005929
	actin cytoskeleton	GO:0015629
	microtubule cytoskeleton	GO:0015630
MSIV-VS-MSVI.DEseq2_Method_F	myosin binding	GO:0017022
	GABA-A receptor activity	GO:0004890
	GABA receptor activity	GO:0016917
	lipid binding	GO:0008289
MSIV-VS-MSVI.DEseq2_Method_P	cellular component assembly involved in morphogenesis	GO:0010927
	actin-myosin filament sliding	GO:0033275
	lipoprotein biosynthetic process	GO:0042158
	autophagy	GO:0006914
	cilium assembly	GO:0042384
	transforming growth factor beta receptor signaling pathway	GO:0007179
	response to transforming growth factor beta	GO:0071559
	cellular response to transforming growth factor beta stimulus	GO:0071560
	cilium morphogenesis	GO:0060271
	lipid metabolic process	GO:0006629
	cell morphogenesis involved in differentiation	GO:0000904
	nonmotile primary cilium assembly	GO:0035058
	cilium movement	GO:0003341
	glycolipid metabolic process	GO:0006664
	spermatogenesis	GO:0007283
	male gamete generation	GO:0048232
	cilium organization	GO:0044782
	response to hormone	GO:0009725
	actin cytoskeleton organization	GO:0030036
	membrane lipid metabolic process	GO:0006643
	cell surface receptor signaling pathway	GO:0007166
	response to lipid	GO:0033993
	gamete generation	GO:0007276
	multicellular organismal reproductive process	GO:0048609
	Kupffer's vesicle development	GO:0070121

	regulation of cell motility	GO:2000145
	cellular lipid metabolic process	GO:0044255
	reproductive process	GO:0022414
	reproduction	GO:0000003
	response to steroid hormone	GO:0048545
	cellular response to hormone stimulus	GO:0032870
	cell motility	GO:0048870
	methylation	GO:0032259
MSV-VS-MSVI.DEseq2_Method_C	cilium	GO:0005929
	lysosome	GO:0005764
MSV-VS-MSVI.DEseq2_Method_F	steroid hydroxylase activity	GO:0008395
	17-alpha-hydroxyprogesterone aldolase activity	GO:0047442
	steroid hormone binding	GO:1990239
	steroid hormone receptor activity	
	estrogen receptor activity	GO:0030284
	hormone binding	GO:0042562
	hormone activity	GO:0005179
	steroid binding	GO:0005496
	methyltransferase activity	GO:0008168
MSV-VS-MSVI.DEseq2_Method_P	C21-steroid hormone metabolic process	GO:0008207
	progesterone metabolic process	GO:0042448
	negative regulation of protein phosphorylation	GO:0001933
	cellular response to hormone stimulus	GO:0032870
	steroid biosynthetic process	GO:0006694
	estrogen receptor signaling pathway	
	steroid hormone mediated signaling pathway	GO:0043401
	response to estrogen	GO:0043627
	hormone-mediated signaling pathway	GO:0009755
	response to hormone	GO:0009755
	cellular response to steroid hormone stimulus	GO:0009725
	cellular hormone metabolic process	GO:0034754
	cellular response to lipid	GO:0034754
	intracellular receptor signaling pathway	GO:0071396
	response to steroid hormone	GO:0030522
	intracellular steroid hormone receptor signaling pathway	GO:0030518
	response to lipid	GO:0033993
	cilium assembly	GO:0042384
	reproductive process	GO:0022414
	reproduction	GO:0000003

	cilium morphogenesis	GO:0060271
	cell motility	GO:0048870
	nonmotile primary cilium assembly	GO:0035058
	hormone metabolic process	GO:0042445
	cilium movement	GO:0003341
	stem cell differentiation	GO:0048863
	lysosome organization	GO:0007040
	lipid metabolic process	GO:0006629
	cilium organization	GO:0044782
	cell surface receptor signaling pathway	GO:0007166
	regulation of hormone levels	GO:0010817
	lipid biosynthetic process	GO:0008610
	Kupffer's vesicle development	GO:0070121
	regulation of cell motility	GO:2000145
	cellular lipid metabolic process	GO:0044255
	regulation of cell proliferation	GO:0042127
	methylation	GO:0032259

Supplementary Table S2. The list of reproduction-related gene family between different developmental stages.

Gene symbol	Unigene name	Species	No. of unigenes	Length	E-value
SPATA1	CL6666.Contig6_All	*Cynoglossus semilaevis*	2	1554	0
SPATA2	Unigene24017_All	*Larimichthys crocea*	2	1626	8.24E-168
SPATA4	CL8276.Contig2_All	*Larimichthys crocea*	4	612	2.13E-83
SPATA5	CL6666.Contig6_All	*Cynoglossus semilaevis*	4	1554	0
SPATA13	CL7784.Contig1_All	*Larimichthys crocea*	2	3774	0
SPATA17	CL4458.Contig1_All	*Larimichthys crocea*	7	291	1.67E-45
SPATA20	CL9502.Contig1_All	*Stegastes partitus*	3	2331	1.50E-10
SPATA22	CL7057.Contig1_All	*Larimichthys crocea*	6	1143	2.86E-144
SPATA45	Unigene5151_All	*Stegastes partitus*	6	258	7.18E-26
BMP1	Unigene491_All	*Larimichthys crocea*	3	2910	0
BMP2	Unigene38765_All	*Paralichthys olivaceus*	9	1266	0
BMP3	Unigene29399_All	*Larimichthys crocea*	1	420	2.17E-72
BMP4	Unigene43791_All	*Oryzias latipes*	1	1320	0
BMP5	Unigene46649_All	*Larimichthys crocea*	5	1428	0
BMP6	CL11848.Contig1_All	*Cynoglossus semilaevis*	2	960	2.70E-179
BMP7	CL11261.Contig1_All	*Larimichthys crocea*	6	594	1.28E-111
BMP8A	CL4358.Contig1_All	*Cynoglossus semilaevis*	1	270	4.37E-38
BMP15	CL4554.Contig1_All	*Stegastes partitus*	3	1356	3.39E-170
BMPR1A	CL11903.Contig1_All	*Larimichthys crocea*	4	1578	0
BMPR1B	CL8156.Contig2_All	*Cyprinodon variegatus*	4	195	1.52E-174
BAMBI	CL6775.Contig1_All	*Dicentrarchus labrax*	2	780	2.84E-142

BMPER	Unigene51103_All	*Stegastes partitus*	2	285	2.76E-48
BMP2K	Unigene22_All	*Stegastes partitus*	6	1914	0
BRINP3	CL5027.Contig1_All	*Stegastes partitus*	3	954	1.20E-180
AQP1	CL4241.Contig2_All	*Hippoglossus hippoglossus*	2	783	1.26E-136
AQP8	Unigene25379_All	*Larimichthys crocea*	2	240	8.60E-39
AQP10	Unigene26904_All	*Larimichthys crocea*	1	216	2.67E-15
AQP11	CL6139.Contig2_All	*Stegastes partitus*	3	807	1.40E-119
SOX1a	Unigene3719_All	*Neolamprologus brichardi*	1	162	2.12E-24
SOX3	Unigene17681_All	*Stegastes partitus*	1	900	1.39E-169
SOX4	Unigene16595_All	*Fundulus heteroclitus*	1	651	1.32E-73
SOX5	Unigene27360_All	*Stegastes partitus*	7	780	1.04E-142
SOX6	Unigene1265_All	*Larimichthys crocea*	1	954	6.52E-179
SOX7	CL10504.Contig1_All	*Larimichthys crocea*	2	996	8.38E-163
SOX8	Unigene20709_All	*Dicentrarchus labrax*	3	1446	0
SOX9	Unigene6266_All	*Scatophagus argus*	2	1131	0
SOX10	Unigene19635_All	*Stegastes partitus*	3	594	1.04E-103
SOX11	CL8588.Contig3_All	*Larimichthys crocea*	11	1113	1.57E-180
SOX13	CL4145.Contig10_All	*Takifugu rubripes*	10	252	1.05E-116
SOX14	Unigene24693_All	*Stegastes partitus*	2	687	9.66E-130
SOX17	Unigene31749_All	*Larimichthys crocea*	1	1200	0
SOX18	Unigene28654_All	*Larimichthys crocea*	1	1812	0
SOX19	CL4306.Contig2_All	*Larimichthys crocea*	2	921	9.34E-174
SOX21	Unigene51476_All	*Pundamilia nyererei*	1	279	4.13E-49

Supplementary Table S3. The list of reproduction-related gene reported before also identified in the transcriptome of turbot between different developmental stages.

Gene symbol	Unigene name	Species	No. of unigenes	Length	E-value
AR	CL3978.Contig2_All	*Cynoglossus semilaevis*	7	930	3.6E-155
CYP	Unigene2885_All	*Larimichthys crocea*	3	306	1.1E-50
FSHR	CL391.Contig67_All	*Takifugu rubripes*	6	285	1.46E-20
GSDF	Unigene31404_All	*Scophthalmus maximus*	2	627	1.95E-116
GDF9	CL4554.Contig1_All	*Stegastes partitus*	3	1356	2.35E-171
MND1	CL8472.Contig4_All	*Cyprinodon variegatus*	1	297	2.00E-19
MAD2	Unigene31372_All	*Poecilia formosa*	2	678	1.12E-107
AMH	CL10501.Contig3_All	*Paralichthys*	1	1494	0

		olivaceus			
STARD5	Unigene16703_All	*Stegastes partitus*	1	633	3.43E-109
STARD7	Unigene699_All	*Stegastes partitus*	1	1224	0
VASA	CL11834.Contig4_All	*Scophthalmus maximus*	7	81	9.52E-08
ZAR1	Unigene34918_All	*Maylandia zebra*	2	1002	7.37E-149
STAR	CL347.Contig2_All	*Lateolabrax japonicus*	2	858	3.07E-156
DMRT	Unigene39319_All	*Paralichthys olivaceus*	8	1248	6.63E-180
ROPN1	Unigene38393_All	*Notothenia coriiceps*	10	684	2.10E-103
SPEF2	CL7543.Contig2_All	*Stegastes partitus*	5	1539	1.43E-171
GnRH	CL10363.Contig2_All	*Thunnus thynnus*	3	291	6.13E-36
ESR1	Unigene5902_All	*Dicentrarchus labrax*	18	1323	4.93E-179
KIF3	CL4298.Contig4_All	*Haplochromis burtoni*	24	879	1.39E-154
KLC3	Unigene16657_All	*Larimichthys crocea*	1	828	3.89E-142
SYCP3	CL8793.Contig1_All	*Acanthopagrus schlegelii*	2	717	1.64E-108
GSK3B	CL9393.Contig2_All	*Stegastes partitus*	2	1275	0
PAQR7	Unigene30680_All	*Paralichthys olivaceus*	2	1062	1.16E-173
PGR	CL4345.Contig3_All	*Larimichthys crocea*	11	987	1.92E-180
20β-HSD	Unigene17505_All	*Larimichthys crocea*	2	300	1.71E-31
MLT	CL6833.Contig1_All	*Stegastes partitus*	10	669	4.02E-120
TSPO	CL9171.Contig2_All	*Larimichthys crocea*	4	483	3.98E-86
GREM1	CL6741.Contig1_All	*Larimichthys crocea*	2	576	7.91E-106

a If more than one unigene was aligned to a single protein, the unigene with the minimum e-value is listed

Supplementary Table S4. The Sm-miR-RNA identified from the transcriptome of turbot by bioinformatics prediction

Name	Sequence
Sm-let-7a-1-3p	CUAUACAAUCUACUGUCUUUC
Sm-let-7b-3p	CUAUACAACCUACUGCCUUCCC
Sm-let-7f-1-5p	UGAGGUAGUAGAUUGUAUAGUU
Sm-let-7f-1-3p	CUAUACAAUCUAUUGCCUUCC
Sm-mir-29b-1-3p	UAGCACCAUUUGAAAUCAGUGUU
Sm-mir-145a-5p	GUCCAGUUUUCCCAGGAAUCCCU
Sm-mir-145a-3p	AUUCCUGGAAAUACUGUUCUUG
sm-mir-199a-1-3p	ACAGUAGUCUGCACAUUGGUUA
Sm-mir-7-3-5p	UGGAAGACUAGUGAUUUUGUUGU
Sm-mir-223-3p	UGUCAGUUUGUCAAAUACCCCA
Sm-mir-143-5p	GGUGCAGUGCUGCAUCUCUGGU
Sm-mir-143-3p	UGAGAUGAAGCACUGUAGCUC
Sm-mir-144-3p	UACAGUAUAGAUGAUGUACU
Sm-mir-138-1-5p	AGCUGGUGUUGUGAAUCAGGCCG
Sm-let-7c-2-3p	CUAUACAAUCUACUGUCUUUCC
Sm-let-7k-5p	UGAGGUAGUAGAUUGAAUAGUU
Sm-mir-199-3-5p	CCCAGUGUUCAGACUACCUGUUC
Sm-let7a-5-5p	UGAGGUAGUAGGUUGUAUAGUU
Sm-let-7b-5p	UGAGGUAGUAGGUUGUGUGGUU
Sm-mir-26b-5p	UUCAAGUAAUCCAGGAUAGGUU
Sm-mir-125a-1-5p	UCCCUGAGACCCUUAACCUGUG
Sm-mir-142a-5p	CAUAAAGUAGAAAGCACUACU
Sm-mir-142a-3p	UGUAGUGUUUCCUACUUUAUGGA
Sm-mir-219-2-5p	UGAUUGUCCAAACGCAAUUCUU
Sm-mir-29b-3p	UAGCACCAUUUGAAAUCAGU
Sm-mir-29a-1-3p	UAGCACCAUUUGAAAUCGGUUA
Sm-mir-142-2-5p	CAUAAAGUAGAAAGCACUAC
Sm-mir-202-1-5p	UUUCCUAUGCAUAUACCUCUUU
Sm-mir-202-1-3p	AGGGGCAUAGGGCAUGGGAAAA
Sm-mir-7-5p	UGGAAGACUAGUGAUUUUGU
Sm-mir-2985-1-5p	GUGGGUGGAAUAGUAUAACAAU
Sm-mir-3074-2-5p	GUUCCUGCUGAACUGAGCCAGU
Sm-mir-3074-5p	GUUCCUGCUGAACUGAGCCAG
Sm-mir-219-5p	UGAUUGUCCAAACGCAAUUCUUG
Sm-mir-3556b-1-5p	ACCGAUUUCAAAUGGUGCUA
Sm-mir-3596d-3p	CUAUACAAUCUACUACCUCA
Sm-mir-3604-1-5p	UAACCAAUGUGCAGACUACUGU
Sm-mir-24-3p	UGGCUCAGUUCAGCAGGAACAG
Sm-mir-4995-5p	AGGCAGUGGCUUGGUUAAGGG
Sm-mir-5368-3p	GGACAGUCUCAGGUAGACA

Sm-mir-21-1-5p	UAGCUUAUCAGACUGGUGUUG
Sm-mir-135b-5p	UAUGGCUUUUUCCUAC
Sm-let-7b-2-5P	UGAGGUAGUAGGUUGUGUGGU
Sm-mir-3064-5p	UGGCUGUUGUGGUGUGCAAAA
Sm-mir-34-5p	UGGCAGUGUCUUAGCUGGUUGU
Sm-mir-1306-5p	CCACCUCCCCUGCAAACGUCCA
Sm-mir-142a-2-3p	AGUGUUUCCUACUUUAUGGAUG
Sm-mir-199c-3p	ACAGUAGUCUGCACAUUGGUU
Sm-mir-2985a-2-5p	GUGGGUGGAAUAAUAUAACAAU
sm-mir-29b-2-5p	CUGGUUUCAGAUGGUGGCUUAG
Sm-mir-125a-5p	UCCCUGAGACCCUU AACCUGUGA
Sm-mir-202-5P	UUCCUAUGCAUAUACCUCUUU
Sm-mir-7-1-3p	CAACAAAUCACAGUCUGCCAAA
Sm-mir-10a-5p	AACCCUGUAGAUCCGAAUUUGUG
Sm-mir-144-5p	GGAUAUCAUCUUAUACUGUAAG
Sm-mir-199-1-3p	AACAGUAGUCUGCACAUUGG
Sm-mir-26a-1-3p	CCUAUUCUGGAUGACUUGGUUC
Sm-mir-142b-5p	CAUAAAGUAGAAAGCACUACUA
Sm-mir-26-1-5p	UUCAAGUAAUCCAGGAUAGGU
Sm-mir-135-1-5p	UAUGGCUUUUUAUUCCUACGUGA
Sm-mir-3556a-5p	CGAUUUCAUAUGGUGCUA
Sm-mir-449-5p	UGGCAGUGUCUUGUUAGCUGGUUG
Sm-mir-2985a-2-3p	UGUUAUAGUAUUCCACCUACCC
sm-mir-181b-1-5p	AACAUUCAUUGCUGUCGCUGGGU
sm-mir-181b-1-3p	CUCACUGAACAAUGAGUGCAA
Sm-let7a-4-3p	CUAUACAGUCUAUUGCCUUCC
Sm-mir-29b-1-5p	GCUGGUUUCAGAUGGUGGCUUAGA
Sm-mir-203-3p	GUGAAAUGUUUCGGACCACUCG
Sm-mir-7-2-3p	CAACAAAUCACAGUCUACCAAA
Sm-mir-34a-3p	CAAUCAGCAAGUAUACUGCCGC
Sm-mir-223-5p	GUGUAUUUGACAAGCUGAGCU
Sm-mir-138-1-3p	GCUACUUCACAACACCAGGG
Sm-mir-21a-5p	UAGCUUAUCAGACUGGUGUUGU
Sm-mir-29c-1-5p	UGACCGAUUUCUUUUGGUGUUC
Sm-mir-10c-5p	AACCCUGUAGAUCCGAAUUUGU
Sm-mir-10c-3p	AAAUUCGUGUCUAGGGGAGAA
Sm-mir-3618-3p	UGUCUACAUUGAUGAAAAGAAC
Sm-mir-34b-3p	AAUCAGCAAGUAUACUGCGCCA
Sm-mir-3533-3p	AUGAAGUGUGACGUCGACAU
Sm-mir-4683-3p	UGGAGAUCCAGUGUUCGCCGGAC
Sm-mir-10a-3p	CAAAUUCGUGUCUAGGGGAGAA
Sm-mir-125a-3p	ACAGGUGAGGUUCUUGGGA
Sm-mir-203-5p	AGUGGUUCUGGACACUUCCACA

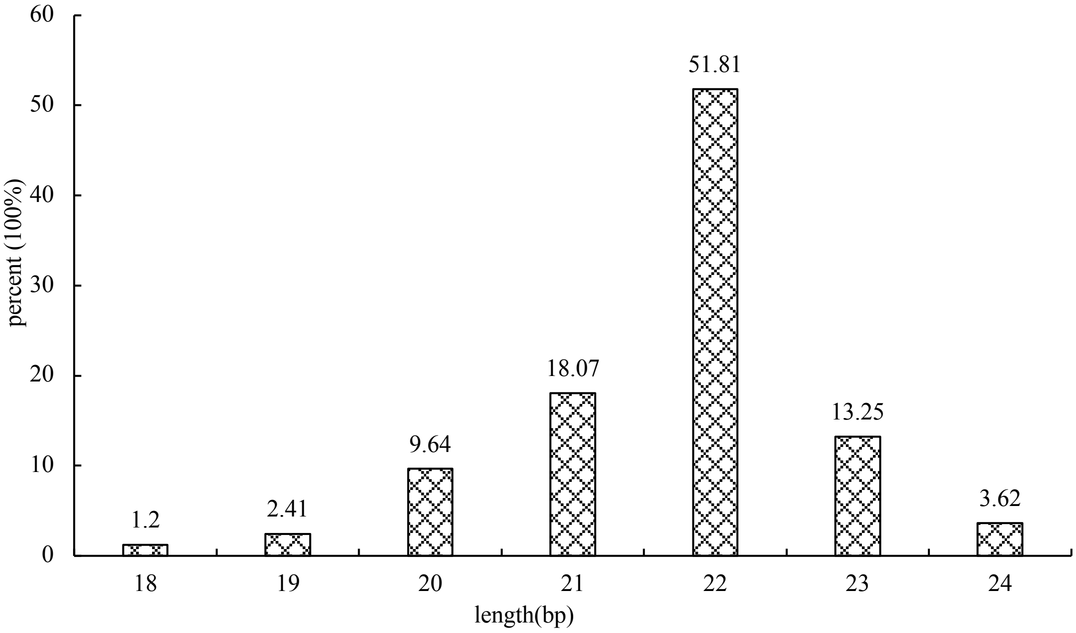

Supplementary Fig. 1 Size distribution pattern of the predicted sm-miRNAs
